# Towards barcode markers in Fungi: an intron map of Ascomycota mitochondria

**DOI:** 10.1186/1471-2105-10-S6-S15

**Published:** 2009-06-16

**Authors:** Monica Santamaria, Saverio Vicario, Graziano Pappadà, Gaetano Scioscia, Claudio Scazzocchio, Cecilia Saccone

**Affiliations:** 1 CNR – Istituto di Tecnologie Biomediche, Sede di Bari, Via Amendola 122/D, Bari, 70126, Italy; 2 Exhicon I.C.T. S.r.l., Via avv. V. Malcangi 254, Trani, 70059, Italy; 3 IBM Italy S.p.A. – IBM Innovation Lab, Via Tridente 42/14, Bari, 70125, Italy; 4 Institut de Gènètique et Microbiologie, UMR 8621 CNRS, Universitè Paris-Sud (XI), Orsay cedex, France; 5 Department of Microbiology, Imperial College London, The Flowers Building, Armstrong Road, London, SW7 2AZ, UK; 6 Dipartimento di Biochimica e Biologia Molecolare "E. Quagliariello", Università di Bari, Via E. Orabona 4, Bari, 70126, Italy

## Abstract

**Background:**

A standardized and cost-effective molecular identification system is now an urgent need for Fungi owing to their wide involvement in human life quality. In particular the potential use of mitochondrial DNA species markers has been taken in account. Unfortunately, a serious difficulty in the PCR and bioinformatic surveys is due to the presence of mobile introns in almost all the fungal mitochondrial genes. The aim of this work is to verify the incidence of this phenomenon in Ascomycota, testing, at the same time, a new bioinformatic tool for extracting and managing sequence databases annotations, in order to identify the mitochondrial gene regions where introns are missing so as to propose them as species markers.

**Methods:**

The general trend towards a large occurrence of introns in the mitochondrial genome of Fungi has been confirmed in Ascomycota by an extensive bioinformatic analysis, performed on all the entries concerning 11 mitochondrial protein coding genes and 2 mitochondrial rRNA (ribosomal RNA) specifying genes, belonging to this phylum, available in public nucleotide sequence databases. A new query approach has been developed to retrieve effectively introns information included in these entries.

**Results:**

After comparing the new query-based approach with a blast-based procedure, with the aim of designing a faithful Ascomycota mitochondrial intron map, the first method appeared clearly the most accurate. Within this map, despite the large pervasiveness of introns, it is possible to distinguish specific regions comprised in several genes, including the full NADH dehydrogenase subunit 6 (ND6) gene, which could be considered as barcode candidates for Ascomycota due to their paucity of introns and to their length, above 400 bp, comparable to the lower end size of the length range of barcodes successfully used in animals.

**Conclusion:**

The development of the new query system described here would answer the pressing requirement to improve drastically the bioinformatics support to the DNA Barcode Initiative. The large scale investigation of Ascomycota mitochondrial introns performed through this tool, allowing to exclude the introns-rich sequences from the barcode candidates exploration, could be the first step towards a mitochondrial barcoding strategy for these organisms, similar to the standard approach employed in metazoans.

## Background

Among the living organisms with the largest effect on human society health and development, Fungi are very widespread. Indeed, man has learned to employ them to produce and transform food, agricultural and industrial resources, drugs and cosmetics or, in certain cases, to fear them as toxic contaminants. In particular, despite the huge progress in feed technology, the contamination of food due to some species, frequently belonging to Fungi, present in natural environment or accidentally introduced during incorrect fabrication or storage procedures, is however possible. In this scenario, the achievement of an effective, rapid and cheap monitoring system of contaminant species to preserve the food quality and foresee possible risks is strongly required. A deeper knowledge of the classification of fungal species and the possibility to discriminate them in an efficient way could strongly support this urgent task. At the same time this is a very challenging mission: Fungi include a broad range of taxa presenting a great variety of morphologies, ecologies and life strategies [1]. Indeed, of the 1.5 millions species belonging to the Fungi kingdom assumed by Hawksworth (1991) [2], fewer than 10% have been formally described. Although Ascomycota harbour a large range of morphologies it is quite difficult to determine distinct and unambiguous species boundaries on the basis of these differences. The difficulty of a morphology-based determination and the wide involvement in human health and life quality of these organisms strongly emphasize the necessity to integrate the classical species identification methods with a taxonomic discrimination system based on DNA [3], a method so rapid and practical to be used easily by both researchers involved in "Fungi species definition challenge" and by non-experts for practical uses. Indeed it is possible to imagine how much this new approach could improve many practical applications, such as the diagnosis of pathogens and invasive species in agriculture or of new species associated to pathological conditions, the identification of dangerous contaminants in food and the revealing of commercial frauds and illegal activities.

At present the possibility to apply DNA barcoding to the identification of fungal species has recently been suggested [3-5]. Moreover new barcode data could provide a definite contribution to fungal phylogeny knowledge even if this kind of research would require integration of additional sequence information [4] (see AFTOL – Assembling the Fungal Tree of Life – initiative for a promising start: ).

Obviously the idea of using a DNA marker to classify taxonomical relationships is not that recent, especially within the Fungi, but, until now, scarce attention has been paid to standardization of the marker to be used. On the contrary, the use of a standard marker is, perhaps, the main innovation of the ambitious DNA Barcode Initiative [6-8], whose aim is to unequivocally identify a species in a particular domain of life, on the basis of a short DNA fragment taken from a standardized portion of the genome [9]. This protocol, potentially very effective and high-throughput in the assignment of unknown specimens to known species, could be applied to all kingdoms of life, being based on DNA, whose language and techniques are universally shared [3]. In general the high degree of standardization promoted by the DNA barcoding approach extends to its entire methodology: the same PCR amplification primers for a defined set of molecular markers would be adopted, the same protocol for sequence data analysis would be used and a common database inclusive of all species barcoded and the same annotations format could be created, allowing also non-specialists to identify species in a fast and cheap way.

Currently, the molecular identification of species in Fungi is based primarily on nuclear DNA markers, such as "nuclear large ribosomal subunit" (LSU rDNA) [10], "nuclear small ribosomal subunit" (SSU rDNA) [11], "internal transcribed spacer (ITS)" [12], "β-tubulin (BenA)" [13], "elongation factor 1-α (EF-1-α)" [14] and "second largest subunit of RNA polymerase II (RPB2)" [15], but the potential use of mitochondrial markers has also been considered due to their favorable features, among which, above all, their high copy number, the possibility of an easier and cheaper recovering of their sequences and the paucity of repetitive regions which could produce misleading results owing to the comparison of non-orthologous sequences pairs. Moreover, the results presented by Seifert et al. (2007) [3], would strongly suggest that a mitochondrial gene could really be a good species molecular marker for Fungi, thanks to its appropriate intra and inter-species variability features. Unfortunately, a serious difficulty in the PCR and bioinformatic surveys is due to the presence of mobile introns in almost all the fungal mitochondrial genes [16]. The aim of this work is to verify the incidence of intron occurrence in Ascomycota, a phylum with a large economic impact both as pests and as beneficial organisms, and to identify one or more mitochondrial gene regions where introns are missing so as to propose them as barcode candidates. In this work we demonstrate that a very effective way to build a map of Ascomycota mitochondrial introns is to extract the information about their positions directly from the annotations of a huge and rich database of nucleic acids, such as Genbank. Reaching this goal is not an ordinary issue since the conventional retrieval system typically used often does not allow fetching specific information included in certain fields of a classical database entry. The design and development of a new query system implemented within a database federation system has allowed overcome the limitation of standard retrieval system. The accuracy of this new tool has been evaluated comparing the results deriving from its use with those obtained from a more classical approach of database similarity searching.

## Methods

The distribution and size of introns in 11 of the 13 protein coding genes and 2 rRNA specfying genes, belonging to Ascomycota mitochondrial DNA, available in public databases, has been assessed using two bioinformatic methodologies: a Blast-based approach and a Query-based approach. ATP synthase F0 subunit 8 (ATP8) and NADH dehydrogenase subunit 4L (NDH4L) genes were discarded from the analysis because we considered them too small compared to the standard size of barcode markers. Indeed in literature the barcodes have generally a size larger than 400 bp whereas the coding region maximum size is 340 bp and 150 bp for NDH4L gene and ATP8 gene, respectively. Designing conserved primers for such small regions would probably produce small amplified regions sizes (probably less than 250 bp for NDH4L gene and less than 50 bp for ATP8 gene) which, even considering some exceptions [26], could have a decreased taxonomic discrimination power. However further analysis is needed to confirm that this assumption is correct for the two genes mentioned above.

### Blast-based approach

The information retrieval system SRS [17], available at the EBI website, has been used for a rigorous extraction of all the mitochondrial Ascomycota sequences from the EMBL database [18] obtained by placing in the searching field "Organelle" the word 'mitochondrion' and in the searching field "Taxon" the word 'Ascomycota'. Then, a Blast similarity searching tool [19] has been applied to the extracted sequences to select orthologous mitochondrial genes, using as probes the sequences of each protein or rRNA coded by the mitochondrial genome of four species scattered across the phylogenetic tree of Fungi: *Aspergillus niger*, *Ustilago maydis*, *Neurospora crassa*, and *Rhizopus oryzae*. In the case of the protein coding genes, the probes used were the amino acidic sequences and the blast procedure was performed using the tblastn algorithm. In the case of the rRNA genes, the probes were nucleotide sequences and the blast protocol used was blastn.

For each gene, the Blast results obtained using the gene probes belonging to all the four species were compared and, in all the cases, it was possible to verify that the results set of at least one search were inclusive of all the sequences found in the others. Thus it was not necessary to integrate the result sets but, simply, to select the most complete one for each gene (see additional file 1 for the final non-redundant list of the EMBL accession numbers of the 4385 sequences obtained with the described procedure and used to perform the following analysis). The local alignment obtained by Blast was used to infer the relative position of the intron sequences for each gene. This approach was based on the assumption that all the nucleotides in the subject sequences lying between two different HSP (High-scoring Segment Pair) need to be considered as introns. In order to control possible errors caused by an excess of divergence among the exon sequences, a python script, designed to score the blast output, performed several control steps before showing the putative presence of an intron. At first, the script would check if the group of HSPs found in a subject sequences, using a given query sequence, were co-linear between query and subject and that they all laid on the same strand on the subject. Furthermore, the script checked if the space between HSPs was present only on the subject sequence, indicating a possible intron, or an equivalent space was found on the query, indicating a stretch of variable sequence. It is known that both blastn and tblastn need a minimum length of similar sequences to detect an HSP (High-scoring Segment Pair), and consequentially small exons may not be detected, but this limitation does not have any influence on the localization of large coding areas free of introns as the ones sought in this work.

### Query-based approach

The task of retrieving an exhaustive set of pieces of information concerning introns of mitochondrial genes in Ascomycota by means of classical query systems leads to non-trivial issues due to both some limitations of these tools and to a quite large and unquantifiable degrees of freedom in annotating records of the primary databases [20]. Especially the latter reason makes difficult to accomplish the research goal exposed in this paper through classical retrieval tools, where functionalities such as term normalization by means of specialized dictionaries, analysis of unstructured information, conditional retrieval and analysis are not available [21,18].

In order to carry out our analysis a hybrid solution has been put in place, in which we coupled the powerful query and retrieval abilities of a relational database with a custom analysis algorithm appositely developed. In this case the relational database used is the LIBI (International Laboratory of Bioinformatics) federated database (LIBI DB) [22]. Although this database has been conceived and set up to solve, within the LIBI platform, the important issue of integrating dislocated and heterogeneous data sources [23], in this work it has been just used as a relational interface to send queries against the primary database GenBank. The LIBI DB has been implemented over the IBM DB2 DBMS (Database Management System) and the IBM WebSphere Federation Server products that allow to access and integrate diverse data and content sources as if they were a single resource, regardless of where the information resides [24].

**Figure 1:**
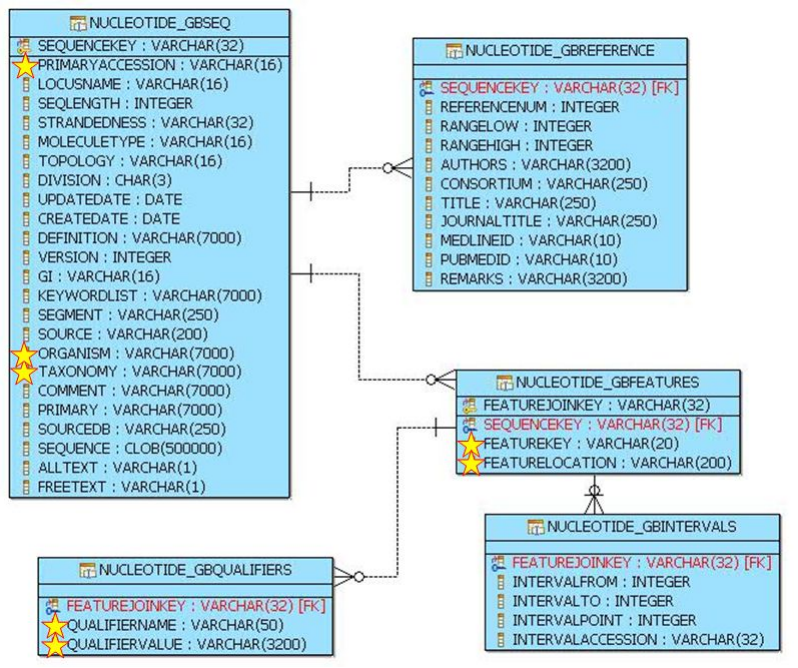
**Sub-schema of the LIBI federated database related to GenBank fields**. Relational representation of the GenBank information as extracted from the overall schema of the LIBI federated database. The fields containing information useful for our analyses are star-marked.

**Figure 2:**
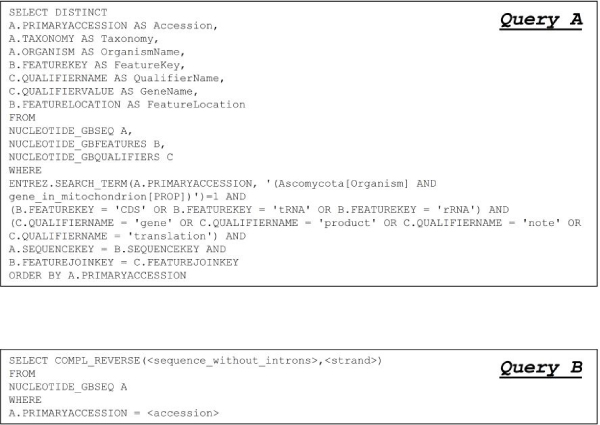
**SQL queries sent against the federated DB to extract information from GenBank**. *Query A *retrieves from GenBank the data useful for the subsequent statistical analysis. The search criteria, included in this query, are shown. They allowed to extract the records related to "Ascomycota" mitochondrial genes containing one term as "CDS", "tRNA", or "rRNA" in the field *feature key *and one of the terms "gene", "product", "note" or "translation" in the *qualifier name *field. *Query B *is used to recover from GenBank the DNA sequences corresponding only to the coding part of each gene.

In our implementation, GenBank federation is achieved through a web-services wrapper interfacing the web-services exposed at NCBI [23]. Fig. 1 shows the relational representation of the GenBank information as extracted from the overall schema of the LIBI federated database. Deducing correspondences between the fields of the relational structure in Figure 1 and those of a GenBank record [25] is straightforward. In the same figure the GenBank fields containing information useful for our analyses are star-marked. These pieces of information are extracted by means of the SQL (Structured Query Language) *Query A *shown in Figure 2. This query involves 3 tables from which values for 7 fields are extracted; the WHERE clause is essentially built upon the criteria of extracting records related to "Ascomycota" as *Organism*, with gene belonging to the mitochondrial genome, that contain one term as {"CDS" -coding sequence-, "tRNA" -transfer RNA-, "rRNA"} in the field *feature key*, and one of {"gene", "product", "note", "translation"} in the field *qualifier name*.

**Figure 3:**
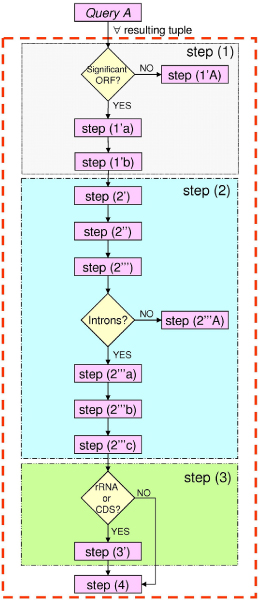
**Custom algorithm used to extract, normalize, clean and prepare data for the subsequent intron evaluation**. The algorithm is composed of four main steps: (1) Analysis of "**qualifier name**" and "**qualifier value**" fields; (2) Analysis of the "**feature location**" field; (3) Analysis of the "**feature key**" field; (4) Creation of a file in which the extracted data are saved as CSV.

Due to both the database "noise" reported before (lack of normalization, poor standardization in feature descriptions, etc.), and complex evaluations to be performed to extract pieces of information relevant for our analysis, a custom algorithm has been designed and implemented in order to evaluate automatically retrieved data. The main steps of the algorithm are depicted in Figure 3 and described in detail below.

The algorithm starts sending *Query A *against the federated database. The retrieved result set is managed by the algorithm in order to obtain refined data organized in a tabular way; this is a suitable format for the statistical analyses conducted later. To each tuple of the result set obtained by *Query A *the four steps (see Figure 3) specified by the algorithm are applied. A tuple is an annotation item retrieved by the SQL query. In this specific case it represents an annotated ORF.

Step (1) analyzes the *qualifier name *and the *qualifier value *fields in order to verify if the tuple refers to a significant Open Reading Frame (ORF). This check is performed evaluating if the *qualifier value *field contains a pattern included in a predefined list of intron gene qualifier (e.g. "intronic ORF", "ORF in an intron", "unnamed protein product", etc.). If the tuple matches a pattern, it is discarded (step 1'A); otherwise the tuple will be handled by step (1'a): this ensures that it is related to a target gene. Step (1'a) evaluates the *qualifier value *field in order to identify the gene name. This piece of information is extracted from the field *qualifier value *corresponding to a *qualifier name *with value "gene" (or "product", if "gene" is missing). In order to avoid gene name synonymy, a suitable dictionary has been used to normalize the gene name the tuple corresponds to (e.g. gene names "coxII" or "cox2", have been normalized to "co2"). Step (1'b) gives also the possibility to extract the protein sequence from the *qualifier value *field (when available) if requested by the analysis.

The whole step (2) is devoted to analyze the *feature location *field. Step (2') infers the correct strand on which the feature lays by checking on the *feature location *for the word "complement". Then, step (2") infers the gene completeness by checking on the presence of special characters (such as "<" or ">", meaning that the start/end sequence position is not defined) in the *feature location *field. Step (2"') evaluates the presence of introns inside the gene and extracts information about them by searching for the word "join" in the *feature location*. If this word is not present, we mark the gene as lacking introns and the algorithm proceeds with the step (2"'A) in order to calculate the start and end position of the gene; if the word "join" is present, the gene contains introns, so the following three steps are performed: step (2"'a) extracts from the *feature location *field the start and end positions for each intron; finally, the start position of the first exon [step (2"'b)] and the end position of the last exon [step (2"'c)] are extracted.

Step (3) is responsible for the analysis of the *feature key *field in order to extract the DNA sequence of the genes specifying rRNA or encoding proteins. So the *feature key *field is checked for the words "rRNA" or "CDS"; if the gene refers to rRNA or CDS a new query, named *Query B *in Figure 2, against the federated database is performed [step (3')] to extract the DNA sequence of the gene without introns. *Query B *is compiled using the SQL user-defined function COMPL_REVERSE we appositely built to complement and reverse the DNA sequence <sequence_without_introns> depending on the annotated strand. In *Query B *the DNA sequence <sequence_without_introns> is reconstructed by means of some of the pieces of information we gathered in the previous steps, while the value of <accession> is that related to the tuple under evaluation.

Finally, step (4) summarizes all extracted information in a file where the data are formatted as comma separated values (CSV).

The performance of such an approach to query GenBank in order to extract relevant information for the analysis of introns in mitochondrial genes in Ascomycota can be effectively evaluated by taking into account some of the quantities reported in Table 1. This table confirms the fact that a custom algorithm, simply constructed over the data extracted by SQL queries sent against the federated database, is suitable to automate the processes of data extraction, normalization and evaluation. Table 1 shows that starting from a large number of tuples such that extracted from the *Query A *(12499) the number of tuples, such as genes, relevant for our study is 11037. The algorithm had been able to extract automatically and correctly all the information contained in the entries' features for about the whole set of the tuples except for 17 of them, for which human examination was requested. However these numbers allow us to state the algorithm accuracy to 99.8%. The 17 records were found in a cross control procedure between assignment proposed by gene name and that suggested by gene description.

**Table 1:** Volumes of tuples managed in this analysis.

**Influenced by step**	**Evaluated quantity**	**Measure**
1	Total tuples in the result set of *Query A*	12499
1'A	Discarded tuples because referring to not significant ORFs	1462
1'a	Total tuples evaluated at step (1'a)	11037
3'	Number of extracted DNA sequences	7234
3	Tuples with not-resolved features	17

The values shown in this table give an idea of the volume of data managed in the retrieval phase of our analysis as well as of the performance level of the approach applied to extract from Genbank the information used for the large-scale analysis of Ascomycota mitochondrial introns.

Finally of the 7234 Genbank entries obtained using the described procedure, 5802 have been selected since they concerned the 13 target genes (11 of the 13 protein coding genes and 2 rRNA coding genes), belonging to Ascomycota mitochondrial DNA, used in this work. See additional file 2 for the final list of the Genbank accession numbers associated to these 5802 entries.

The developed query system at present is not publicly available, however we are evaluating a solution to make it available as a service that researchers could use through the internet.

## Results

### Introns position and size by Blast-based approach

**Figure 4:**
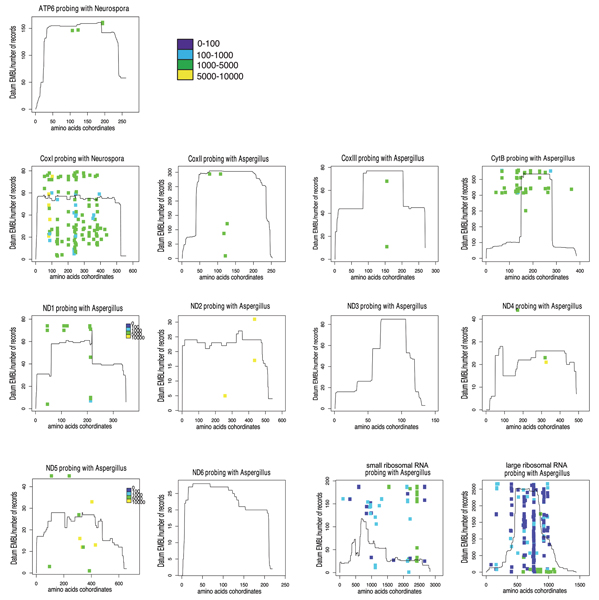
**Intron map of Ascomycota mitochondrial genes by the Blast-based approach**. The intron positions along each Ascomycota mitochondrial gene, revealed by the Blast-based approach, are blotted. The coloured dots show the insertion positions and size (hue of colour) of putative introns. The height of the profile indicates the number of EMBL records that matched with each site of the probe. The introns positions are relative to the sequence of one of the four reference species probes used. In particular the species for which the results set included all the sequences found with all the other three probes (see the "methods" section for the details).

The results of the Blast-based approach, shown in Figure 4, reveal the pervasiveness of introns across almost all the Ascomycota mitochondrial genes included in the analysis, namely cytochrome oxidase subunit I (CO1), cytochrome oxidase subunit II (CO2), cytochrome oxidase subunit III (CO3), cytochrome b (CYTB), ATP synthase F0 subunit 6 (ATP6), NADH dehydrogenase subunit 1 (ND1), NADH dehydrogenase subunit 2 (ND2), NADH dehydrogenase subunit 4 (ND4), NADH dehydrogenase subunit 5 (ND5), large subunit ribosomal RNA (lRNA) and small subunit ribosomal RNA (sRNA), even if the distribution density and the size of these non-coding regions can vary remarkably between them. Particularly CO1, CYTB and the two ribosomal genes show the maximum density of introns. According to these results, the CO1 gene, designated as the core barcode region for animals, has to be reconsidered in Fungi, due to the potential complication in PCR-based surveys related to the massive presence of introns, as observed by Seifert et al. in 2007 [3]. On the other hand, at least three other protein coding genes, or part of them, could be reasonably considered barcode candidates because of their scarcity of introns, namely ND3, ND4 and ND6. The ND3 gene, with a length of approximately 400 bp, seems to be too short to be considered as a barcode marker however, several recent studies demonstrated that reducing species marker length has a profound effect on the accuracy of the resulting phylogenetic trees, but surprisingly the species discrimination is still effective [4,26].

**Figure 5:**
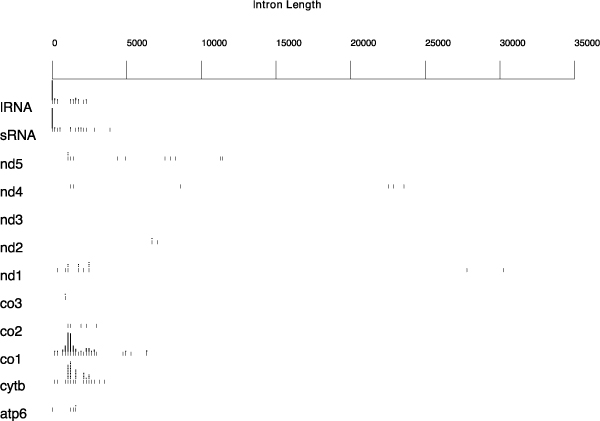
**Intron size distribution as estimated by the Blast-based protocol**. The distribution of size in each gene (genes acronyms are marked on the left size of the graph) is shown with a so-called "barcode graph", in which each small vertical bar represent the size value of a given intron and each dot on the top of the vertical bar represent further introns which have the same size. From the distribution of intron sizes one record (AY955840 and its equivalent from full genome collection NC_007935) was removed because its size value was too large (>50000 bp) to be easily drawn on the graph.

Looking at the distribution of intron sizes (The results of our analysis are shown in Figure 5), the values range between 500 and 3000 bp, with few exceptions, the median value is 1269 and the estimated mode is 1240.457. An intrinsic problem of the Blast-based approach, which is based on the recognition of sequence similarity (the similarity score between the probe and each sequence in the database depends on the fraction of identical residues and on the lengths of the matching regions), is that small exons could not be identified and several introns could be joined together producing erroneous results, as shown in Figure 5 where unrealistic introns about even over 25000 bp emerge. For this reason, a query-based approach seemed to be immediately desirable in order to calculate the intron positions and sizes directly from database annotations.

**Figure 6:**
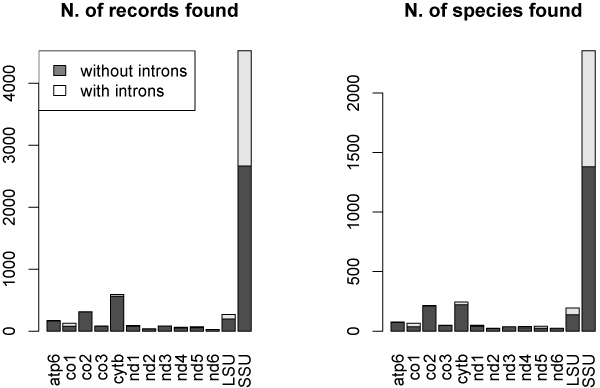
**Number of records and species found with and without introns using the Blast approach**. Histogram representing the number of a) records and b) species found with the Blast approach for each of the selected genes. Each bar is split in grey and white zones proportionally to the number of records without any introns or with introns, respectively.

A summary of number of records and different species retrieved for each gene is displayed in Figure 6.

### Introns position and size by Query-based approach

**Figure 7:**
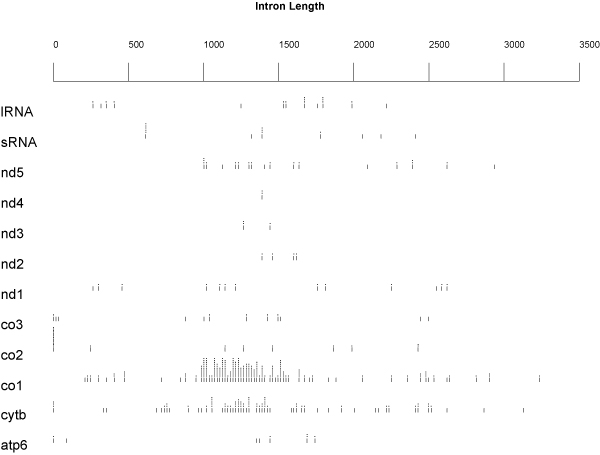
**Intron size distribution as estimated by the Query-based protocol**. The distribution of size in each gene are depicted as for the blast protocol results. From the distribution of intron sizes one record (AY955840 and its equivalent from full genome collection NC_007935) was removed because with a value too large (14969 bp) to be easily recorded on the graph.

**Figure 8:**
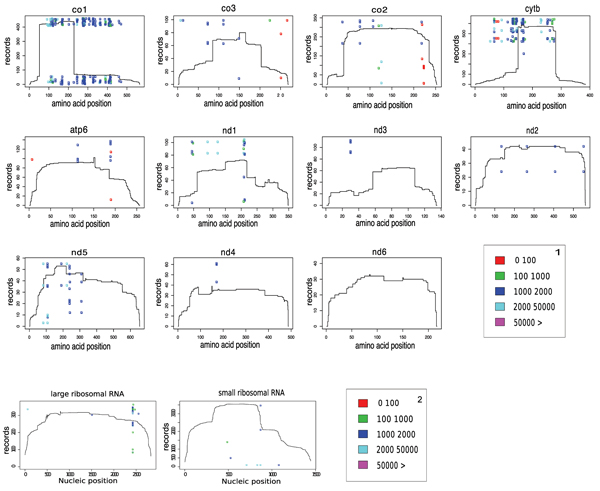
**Intron map of Ascomycota mitochondrial genes by the Query-based approach**. The intron positions along each Ascomycota mitochondrial gene, revealed by the Query-based approach, are blotted. The coloured dots show the insertion positions and size (hue of colour) of introns. The height of the profile indicates the number of records, extracted from Genbank, that matched with each site of a certain CDS or mature rRNA (see the "methods" section for the details).

**Figure 9:**
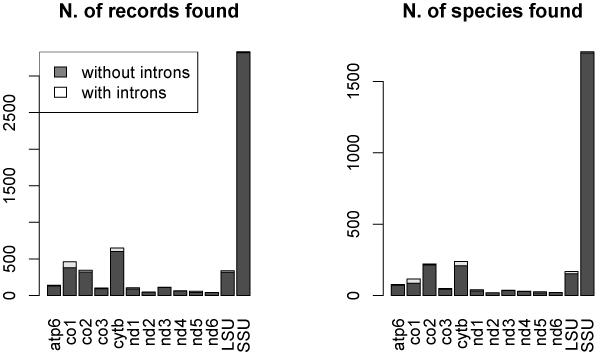
**Number of records and species found with and without introns using the Query approach**. Histogram representing the number of a) records and b) species found with the Query approach for each of the selected genes. Each bar is split in grey and white zones proportionally to the number of records without any introns or with introns, respectively.

The Query-based approach generally confirmed the results obtained with the Blast-based one, but it seems more accurate in recognizing also small exons (almost the totality of intron sizes are lower than 3500 bp). Indeed, as shown in Figure 7, the intron sizes are still included in a range between 500 and 3000 bp, with few exceptions. The majority of them are found in a range between 1000 and 2000 bp and the unrealistic intron sizes revealed by the Blast approach are not found any longer. Figure 8 displays that CO1 and CYTB genes show again a high density of introns. As for the rRNA genes, the situation is not clear: the large amount of introns highlighted through the Blast method disappeared almost completely as a result of the Query-based method. Therefore, further investigation of the ribosomal RNA genes is required. The ND3 gene does not appear to be completely intron-free as found with the Blast-based approach. Finally, even if several Ascomycota mitochondrial genes show a low incidence of introns, such as ND3, ND4 and the terminal 3' of ND5, ND6 appears to be completely intron-free and thus it constitutes the best potential barcode candidate. A summary of a number of records and different species retrieved for each gene is displayed in Figure 9, respectively. See additional file 3 for the original data about the presence and the position of introns in the totality of mitochondrial Ascomycota records present in Genbank used to obtain the graphs reported in Fig. 8.

## Discussion

The study described here has allowed an evaluation of the impact of the mitochondrial introns in the development of a mitochondrial barcode system for the molecular discrimination of Ascomycota species. At the same time, an accurate methodological test, in which two alternative bioinformatics approaches to reveal intron size and position has been performed. The Query-based approach, developed as part of a database federation system, including public (such as Genbank) and private resources, has allowed to characterize precisely the intron position and size, performing a data retrieval directed only to obtain specific information usually present in the entries of a biological database, but hardly selectable through the classical query systems. Indeed, the commonly used databases query systems such as SRS () or Entrez , although giving the possibility to carry out complex research in which several criteria are combined and the false positives minimized, appear rather ineffective when it is necessary to retrieve selected information contained in particular fields of the entries. This problem is particularly evident when we wish to extract the data included in the so-called Feature Tables which, in a typical entry of a nucleic acids database, contain a detailed description of the various functional and structural portions of the sequence. The new query system, implemented in the LIBI platform, has been very useful for a rigorous extraction of only introns start and end positions data, present in the lines of the Feature Tables and it has allowed focusing of the query only on the mitochondrial entries of the Ascomycota annotated in Genbank.

The Blast approach provided only an approximation of the intron position localization but it has the advantage to be independent from the database entry annotations which could be incorrect or absent. Then, even if the query approach seems most accurate in determining the exact start and end intron positions, we should also consider the results obtained using the Blast approach.

The results obtained from both the Blast and Query methodologies confirm the high frequency of mobile introns [16] in almost all the mitochondrial genes of Ascomycota indicating that only few genes, primarily the ND6 gene, seem not plagued by the presence of introns and could be reliably amplified to be successfully used as barcode species markers in this phylum. On the basis of this pure bioinformatic analysis, beside the ND6 gene, the ND3 gene and the terminal 3' of the ND4 and ND5 genes, appear to be good barcode candidates due to the scarcity of introns in a considerable part of their sequence. Nevertheless further tests are indispensable to evaluate the possibility to design pairs of conserved primers, working in a wide taxonomic range, that would produce an amplified fragment which still harbours enough species discrimination power.

According to the results obtained performing the query approach, also the two rRNA genes contain some large regions (until 2400 bp long in lRNA and 500 bp long in sRNA) free from introns which could be considered good barcode candidates but, in this case, the results of Blast approach strongly contradict this observation requiring further investigations to understand if the intrinsic frame shift mistakes in the rRNA nucleotide alignment performed by Blast lead to false interruptions of the CDS, as strongly suggested by the dimensions of the majority of introns (<100 bp).

The comparison of the two approaches used in the analysis presented above show a higher efficiency of the Query-based system, giving that it aims directly to the information annotated in the database and avoids a series of alignment and retrieval stages based on the similarity calculation which inevitably produce false positives and negatives in the final results. In conclusion we were not able to claim that the Blast approach can be a watchdog of the query approach, as illustrated by the case of the huge number of introns found in the large and small ribosomal RNA genes and the problem of excessive intron length in the protein coding genes shown in Fig. 5 but expect that in some occasions Blast could point out some annotation errors.

The idea of using a mitochondrial gene as a barcode marker in Fungi, just as it adopted in animals, derives from several simple considerations. First of all, the general favorable features of mitochondrial DNA, suitable for the role of a taxonomic marker, have to be considered. Among them, above all, there are the high copy number, the possibility of an easier and cheaper recovering and the paucity of repetitive DNA which makes the assessment of the homology, both at the level of loci and at the level of a single site, quite simple. Indeed comparative studies on mitochondrial coding genes are not plagued by doubts on the orthology of a particular locus compared between different samples as it could occur for nuclear genes, although the accidental amplification of nuclear mitochondrial (NUMTs) pseudogenes could lead to problems [27-29]. In addition the good results obtained in the animal kingdom, where a region of the CO1 mitochondrial gene of about 650-bp provides a high resolution to discriminate species in almost 95% of taxa belonging to various animal lineages [30-32], encourage to extend the test of the same marker also to the other kingdoms, including Fungi. This aspect becomes extremely noteworthy if we consider the barcode Initiative efforts towards a high level of protocols standardization [33]: to use a universal barcode gene to identify species in all kingdoms would be ideal for DNA barcoding, producing many benefits thanks to the subsequent improvement of rapid, simple and inexpensive applications for both researchers involved in basic biodiversity studies and non-expert interested in several practical issues. Two important questions about the evolutionary dynamics of the mitochondrial genes immediately arise. The first one concerns the commonly accepted idea that a good barcode should exhibit sufficient variability to allow discrimination between species and low variability within individuals belonging to the same species [34]: could a single gene sequence, selected as a barcode marker, have the appropriate molecular evolution dynamics along the evolutionary lineages of an entire kingdom? The second question is: can the variability features of a certain marker be comparable among distant taxa, such as those belonging to separate kingdoms, or different dynamics have to be considered when the genetic distances are calculated and compared? These could seem commonplace questions, but they acquire an urgent and crucial sense in the scenario of a fast developing initiative, such as the DNA barcode, whose ambition is a very large scale discrimination of world biodiversity at the species level. Indeed, we don't know yet if evolutionary dynamics of mitochondria are radically different between Fungi and metazoa and we don't know if the profound difference between these organisms in life style and reproduction mode could be reflected in their evolutionary dynamics and, if so, in which way [35]. In the case of metazoans, the good species discrimination capacity of mitochondrial CO1 gene could be foreseen on the basis of some theoretical considerations and previous experimental analysis [36-38], but it is difficult to use the same theoretical argumentations in Fungi above all given the latter very different reproduction system. Some recent data suggest that, despite these differences between animals and Fungi, the patterns of barcode variation are quite comparable between the two groups. In particular the 5' end of the CO1 gene provided an excellent resolution at the species level also in Fungi [3,4]. Two important problems disturb the use of CO1 or other mitochondrial sequences as barcodes in this group of organisms. First, there are only five protein-coding genes (CO1, CO2, CO3, CYTB and ATP6) common in all fungal mitochondrial genomes available [4]. Obviously this statement reduces consistently the candidates list to be investigated for the role of "universal barcode" suitable for all Fungi taxa and, in particular, it excludes just the genes which, according to our analysis in Ascomycota, are less pervaded by introns. The presence of mobile introns itself represents the other serious difficulty concerning the use of a mitochondrial gene as barcode unless an alternative laboratory methodology is taken in account, such as the amplification of mRNA using RT-PCR [39] or the use of COI sequences in particular members of some taxa, such as the Penicillium subgenus Penicillium, which has an unusual scarcity of introns, indicating that a small fraction of CO1 sequences could be stripped of introns for part of their life cycle [3]. Unfortunately this would result in both a decrease of easiness and quickness of the protocol and a strong limitation of the field of action of barcode from the point of view of taxonomic range and life-stage which could be analysed. Our analysis suggests that the intron problem in the CO1 gene should not be underestimated and many tests have to be performed in different genera of Fungi to obtain more general rules and common solutions.

In conclusion, it seems difficult to identify a "universal barcode" able to resolve species in all Life, even if encouraging results come from the numerous studies in animals, where the CO1 gene seems successful in many taxonomic groups [34,40]. In Fungi the effectiveness of an approach including a single species-level molecular marker has to be verified yet. This concerns both mitochondrial and nuclear candidates, indeed several studies have demonstrated that each candidate could work correctly in definite species ranges, while being quite ineffective in other ones [41,42,3]. Our preliminary studies concerning the power of mitochondrial ND6 gene to discriminate species in Fusarium, a genus belonging to Ascomycota phylum, confirm this trend (data not shown) and suggest that designing a multi-locus approach where mitochondrial and nuclear markers were integrated could really help to reach a profound discrimination of the terminal nodes of fungal phylogeny.

## Conclusion

The Barcode of Life Initiative represents an innovative resource for the world biodiversity knowledge at the level of its basic units: the species. This original and emergent system is opening the way to new and inspiring applications both in basic research and practical issues due basically to its high degree of standardization. The huge amount of data which have to be produced and analysed along this massive project unquestionably requires a strong bioinformatics support and the improvement of experimental and practical methodologies working effectively on a large scale. A contribution in this sense could be given by the development of a new query system able to extract and manage specific information, hardly selectable with the classical retrieval systems, from Genebank. In particular, this system has been applied to generate a very accurate and detailed map of Ascomycota mitochondrial genome introns in order to recognize one or more regions, free or poor of introns, which could be easily used as barcode markers in this phylum. Indeed, the non-coding sequences interrupting quite all the Fungi mitochondrial genes could represent one of the most serious difficulties in amplifying and analysing a taxonomic molecular marker. The encouraging results obtained testing this new bioinformatic query tool suggest that it could represent a preliminary step towards a mitochondrial barcoding strategy for Fungi, similar to the standard approach routinely employed in metazoa, allowing to choose as barcode candidates those mitochondrial genome regions not perturbed by the "intron problem".

## Competing interests

The authors declare that they have no competing interests.

## Authors' contributions

MS participated in the design and interpretation of data of the Blast-based study, in the conceiving and development of the Query-based analysis protocol and in analysis and interpretation of its data and drafted the manuscript. SV designed and developed the Blast-based study, participate in the conceiving of the Query-based protocol and to the analysis and interpretation of its data, and help to draft the manuscript. GP participated in design and development of the Query-based approach and helped to draft the manuscript. GS participated in design and development of the Query-based approach and helped to draft the manuscript. CS (Claudio Scazzocchio) participated in the conceiving of the study and revised critically the manuscript. CS (Cecilia Saccone) participated in the conceiving and coordination of the study and revised critically the manuscript.

## Supplementary Material

Additional file 1 **List of the EMBL entries obtained through the Blast-based search**. This file contains the final non-redundant list of the EMBL accession numbers concerning the 4385 sequences obtained from the comparison of the results sets produced by Blast procedures in which the sequences of each protein or rRNA coded by the mitochondrial genome of four species, such as *Aspergillus niger*, *Ustilago maydis*, *Neurospora crassa*, and *Rhizopus oryzae *are used as probes. The alignments between the EMBL sequences and the probes, during the Blast runs, allowed to picture the introns map of the 13 Ascomycota mitochondrial genes included in this work. Click here for file

Additional file 2 **List of the Genbank entries retrieved through the Query-based search**. This file contains the final non-redundant list of the Genbank accession numbers related to the 5802 entries obtained and further analysed through the new Query-system described in this work, with the aim to read the introns positions and length directly from the Feature Tables. Click here for file

Additional file 3 **Intron map of Ascomycota mitochondrial genes as retrieved in the query-based analysis**. The table summarize the presence and the position of introns in the totality of mitochondrial Ascomycota records present in GenBank. Each row describe a record without introns or with an intron and for each one the following information, were applicable, were add: "AC": GeneBank accession number; "Gene.Name": standardized locus name; "Intron.Start": intron starting position relative to the start of the CDS in the record; "Intron.End": intron ending position relative to the start of the CDS in the records; "Abs.Intron.Start": intron starting position relative to the start of the whole record; "Abs.Intron.End": intron ending position relative to the start of the whole record; "Insertion.point.in.Ref": intron insertion position mapped on the protein of the reference organism; "L.intron": length of the intron. Each row is identified with a name composed by the accession number, the standardized locus name and an increasing number to distinguish more than one intron present in the same CDS. Click here for file
